# Context-Aware Diagnostic Specificity (CADS)

**DOI:** 10.3390/bios12020101

**Published:** 2022-02-07

**Authors:** Eric S. McLamore, Geisianny Moreira, Diana C. Vanegas, Shoumen Palit Austin Datta

**Affiliations:** 1 Department of Agricultural Sciences, Clemson University, Clemson, SC 29634, USA; 2 Global Alliance for Rapid Diagnostics, East Lansing, MI 48824, USA; geisiam@clemson.edu (G.M.); dvanega@clemson.edu (D.C.V.); 3 Biosystems Engineering, Department of Environmental Engineering and Earth Sciences, Clemson University, Clemson, SC 29631, USA; 4 MIT Auto-ID Labs, Department of Mechanical Engineering, Massachusetts Institute of Technology, 77 Massachusetts Avenue, Cambridge, MA 02139, USA; shoumen@mit.edu; 5 MDPnP Interoperability and Cybersecurity Labs, Biomedical Engineering Program, Department of Anesthesiology, Massachusetts General Hospital, Harvard Medical School, 65 Landsdowne Street, Suite 232, Cambridge, MA 02139, USA

## Commentary

Rapid detection of proteins is critical in a vast array of diagnostic or monitoring applications [1,2,3]. Development of point of need (PON) devices is expanding rapidly, facilitated by technological improvements in miniaturization and enhanced access to scalable manufacturing equipment [4]. Examples of PON devices include detection of exposed proteins on pathogens, (e.g., SARS-CoV-2 spike protein [5,6]), detection of protein biomarkers [7,8] (e.g., cancers, cardiac diseases, autoimmune diseases, and acute events), and identification of plant pathogens in the food system (e.g., *Listeria* spp. invasion proteins [9,10]). In this special issue of Biosensors, we build on the concept of context-awareness through establishment of CADS: Context-Aware Diagnostic Specificity, an application specifically focused on diagnostics. The notion of *context awareness* is a computational discovery framework [11], and has recently been extended for applications in cell-cell communication [12], biological inspired design [13], and synthetic biology [14]. For application in diagnostics, context awareness is extended to include molecular systems applied for specific detection of a target (or multiple targets). Here, the term specificity is used broadly and also includes elements of selectivity and accuracy, as discussed by Peveler et al. [15]. CADS requires convergence of sensor engineering/science (from molecular scale to device and system scale [16]) with the domain of public health and natural resources (spanning food-water-sanitation-health systems). 

Research on engineering molecular systems to optimize affinity between receptor(s) and target molecules is the most granular aspect of diagnostic sensing. Molecular events must also be scaled to the device level, where techniques such as embedded controls and multiplexing [17] may be necessary for monitoring complex targets such as proteins. Data acquired from diagnostic tools must be analyzed and features extracted for communication with the user (without losing the granularity of the data). Analyzed sensor data must also merge with the public health and food/water system using ethical and socially-responsible decision support framework(s) [18]. Thus, decision science acts as a bridge between these domains, connecting digitized sensor data with socioeconomic and cultural factors for ensuring access and connectivity with the aim of reducing environmental risks, improving food systems, and mitigating health impacts.

The current pandemic is an example of the development of devices focused on public health for clinical diagnosis of Severe Acute Respiratory Syndrome Coronavirus 2 (SARS-CoV-2) within the concept of CADS. The S-protein-ACE2 binding has been the model of study for molecular interactions and development of both rapid diagnosis tools [19] and screening of potential inhibitors [20]. To date, based on the specificity of S-protein-ACE2 interactions impedimetric-based biosensing platform has the potential for rapid diagnosis of SARS-CoV-2 infections in a limit of detection range from femtogram to nanogram in clinical human saliva samples [21,22]. Beyond diagnostic device development, a convergence with data science to translate the output in a friendly-understandable user interface such as a smartphone readout [23] is of utmost need within the CADS spectrum. The “context” of the SARS-CoV-2 viral load in asymptomatic, pausi-symptomatic and uninfected vs infected states are important factors which are likely to infuence the outcome of the molecular diagnostics system. SARS-CoV-2 also has a spatio-temporal context with respect to origin of the test specimen because the distribution of the virus varies with nasal, nasopharyngeal, upper respiratory tract and mucosal (sputum) samples. These issues highlight the complexity of the context and the need for awareness (beyond the diagnostics domain). Continuing this example, the CADS concept calls for data from diagnostic tools to fuse with other context-aware devices such as proximity sensors for location tracking [24]. Altogether, advances in biosensor diagnostic platforms for SARS-CoV-2 coupled with metadata (e.g., sample location, contact tracing) may inspire and amplify the efforts towards food and water systems domain.

The transition of a biosensor technology from a laboratory prototype to a finished diagnostics tool in the hands of users can be a intrincate process that most researchers have not succesfully navegated, as demostrated by the disproportionate ratio between the large number of “novel” biosensors published in the scientific literature in the past decades, and the compartively low number and variety of biosensing tools available to the public, particularly for non-clinical applications. This situation is unfortunate because diagnostic tools that can be deployed outside of clinical settings, such as in remote rural communities, are still urgently needed.

CADS intentionally casts a wide net in an attempt to capture transdisciplinary research that aims to advance the field of diagnostic biosensors. Research focused on more than one aspect are sorely needed in order to drive convergence toward meaningful outcomes. 
